# Therapy targets in glioblastoma and cancer stem cells: lessons from haematopoietic neoplasms

**DOI:** 10.1111/jcmm.12122

**Published:** 2013-09-02

**Authors:** Maria Linda Cruceru, Monica Neagu, Jean-Baptiste Demoulin, Stefan N Constantinescu

**Affiliations:** a Department of Cellular and Molecular Medicine, Carol Davila University of Medicine and PharmacyBucharest, Romania; b Immunobiology Department, National Institute of Pathology “Victor Babeş”Bucharest, Romania; c Biochemistry–Proteomics Department, POSCCE CANBIOPROT at National Institute of Pathology “Victor Babeş”Bucharest, Romania; d MEXP Unit, de Duve Institute, Université catholique de LouvainBrussels, Belgium; e Signal Transduction and Molecular Hematology Laboratory, Ludwig Institute for Cancer ResearchBrussels; f Cell Signaling Unit, de Duve Institute, Université catholique de LouvainBrussels, Belgium

**Keywords:** glioblastoma cancer stem cell, cell surface markers, signalling pathways, haematopoietic stem cells, leukaemia

## Abstract

Despite intense efforts to identify cancer-initiating cells in malignant brain tumours, markers linked to the function of these cells have only very recently begun to be uncovered. The notion of cancer stem cell gained prominence, several molecules and signalling pathways becoming relevant for diagnosis and treatment. Whether a substantial fraction or only a tiny minority of cells in a tumor can initiate and perpetuate cancer, is still debated. The paradigm of cancer-initiating stem cells has initially been developed with respect to blood cancers where chronic conditions such as myeloproliferative neoplasms are due to mutations acquired in a haematopoietic stem cell (HSC), which maintains the normal hierarchy to neoplastic haematopoiesis. In contrast, acute leukaemia transformation of such blood neoplasms appears to derive not only from HSCs but also from committed progenitors that cannot differentiate. This review will focus on putative novel therapy targets represented by markers described to define cancer stem/initiating cells in malignant gliomas, which have been called ‘leukaemia of the brain’, given their rapid migration and evolution. Parallels are drawn with other cancers, especially haematopoietic, given the similar rampant proliferation and treatment resistance of glioblastoma multiforme and secondary acute leukaemias. Genes associated with the malignant conditions and especially expressed in glioma cancer stem cells are intensively searched. Although many such molecules might only coincidentally be expressed in cancer-initiating cells, some may function in the oncogenic process, and those would be the prime candidates for diagnostic and targeted therapy. For the latter, combination therapies are likely to be envisaged, given the robust and plastic signalling networks supporting malignant proliferation.

- Introduction
- Cancer stem/initiating cells
  – Analogies with blood myeloid malignancies of different grades
  – Cancer stem cells in tumours with different grades of malignancy
  – Cancer stem cell microenvironment – relationship with niche
- Markers associated with tumour-initiating cells
  – CD133- as cancer stem-cell marker
  – Hand-in-hand CD133 and nestin expression
  – Musashi-1 anti-differentiation regulator
  – Other markers
- Signalling pathway deregulation
  – Receptor tyrosine kinases, downstream PI-3′-kinase (PI3K) and Akt
  – Notch
  – TGF-b and PDGF
  – Bone morphogenetic proteins
  – Sonic Hedgehog and Wnt
- STAT3 and STAT5
- Epigenetic alterations in gliomas: analogies with human myeloid cancers
  – Bmi-1
  – IDH1, IDH2 and TET2 mutations
- Concluding remarks

## Introduction

Cancer stem cells were identified in a variety of human solid tumours, including brain tumours [1–4], prostate tumours, pancreatic adenocarcinomas, colon carcinomas, hepatocellular carcinomas, melanomas, breast cancers, lung cancers, laryngeal carcinomas and osteosarcomas [5], as well as blood cancers, such as acute leukaemia and chronic conditions like human myeloproliferative neoplasms (MPN) [6]. The field has been inspired by advances in the study of blood formation from haematopoietic stem cell (HSC) and cancer-initiating cells in leukaemia [7–9]. Based on this paradigm, intense research is unfolding in several extremely aggressive tumours to identify the cancer-initiating cells. More recently, a distinction is being made between cancer-initiating cells and cells that allow propagation of the tumour [10].

WHO classification of central nervous system (CNS) tumours includes astrocytic (with glioblastoma multiforme -GBM- or grade IV astrocytoma being the most aggressive), oligodendroglial, mixed oligoastrocytic malignant diffuse gliomas or ependymal tumours [11]. The characteristics that are taken into account when grading gliomas are tumour cell mitotic index, nuclear atypia, vascular proliferation and necrosis [12]. Tumours with well-differentiated cells, biologically benign and curable are considered grade I; grade II gliomas belong to a stage that associates with long clinical courses, this stage, however, is incurable by surgery because of early diffuse infiltration of tumour cells in the surrounding brain tissue; tumours showing increased anaplasia and tumour cell proliferation, being more rapidly fatal, are classified as grade III; while grade IV gliomas or astrocytoma (glioblastoma multiforme) display the highest degree of malignancy, particularly vascular proliferation and necrosis. This stage is lethal within a mean survival of 1 year. Summing up, grade I and grade II gliomas are considered low-grade, while III and IV are high-grade, or highly malignant tumours [12].

In Europe, the incidence of primary CNS cancers ranges from 4.5 to 11.2 cases per 100,000 men and from 1.6 to 8.5 per 100,000 women. Astrocytic tumours are the most common tumours (86.0%), of which 63% are highgrade [13].

The discovery of glioma cancer stem cells (GCSCs) has offered an explanation for several clinical challenges regarding aggressiveness, relapse and treatment resistance [14]. This concept is still intensely debated in other cancer areas, such as cutaneous melanoma [15, 16]. The debate has been focusing on whether certain tumour cells exhibit, at a certain moment, cancer-initiating activity, or whether they represent the particular minority of the tumour that perpetuates cancer. Recent articles have focused on cancer stem-cell involvement in initiation, progression and recurrence of brain tumours and their clinical and biological characterization [5, 17–20].

Despite the intense work performed on cancer stem cells in malignant brain tumours, it was only in the last years that stem cells, regarded also in the context of low-grade tumours such as grade I astrocytoma, have developed into a new research area [21]. Thus, in this scenario, any tumour, even low-grade or benign, needs a reservoir of initiating cells. A major speculation in the field has been that mechanisms that control the quiescence and reservoir of normal tissue stem cells might be similar in cancer stem cells. This view seems to be supported by recent exquisite studies where transcriptional profiles were examined in colon tumours exhibiting heterogeneity with respect to single cell gene expression profiling. In these experiments, it was demonstrated by single cell PCR that gene expression of different transcriptional identities mirrors those of different cellular lineages of normal colon [22]. Furthermore, in leukaemias like chronic myeloid leukaemia (CML), effective treatment with imatinib leads to undetectable levels of BCR-ABL; however, recent evidence suggests that CML stem cells are insensitive to kinase inhibitors and responsible for minimal residual disease in treated patients [23]. Imatinib is a type II (binds to the inactive state kinase) adenosine triphosphate competitive inhibitor of Abl (and Bcr-Abl), KIT and platelet-derived growth factor receptor (PDGFR) kinases, one of the first cancer-targeted therapies [24].

The focus of our review is to present signalling pathways and proteins that appear essential for initiating, maintaining and propagating the malignant phenotype. In certain cases, inhibitors of those pathways exist, and the clinical attempts to use single pathway or receptor inhibitors did not lead to effective treatment. Thus, combination therapies are likely to be envisaged once the precise mechanisms and pathways operating in cancer initiating cells will be determined.

## Cancer stem/initiating cells

More than 200 years ago, the father of pathology, Rudolf Virchow, suggested that cancer cells arise from embryonic-like tissue (Virchow, 1858). Nowadays, the power of genomics and epigenetic techniques led to the notion that indeed cancer hijacks genetic and epigenetic specific programmes from embryonic development circuits.

There are two models regarding the generation and function of GCSCs, the stochastic and the hierarchical models [25]. In the stochastic model, GCSCs are cancer stem cells derived from committed or differentiated cells, which acquire genetic mutations towards immortal proliferative capacity. In contrast, the hierarchical model suggests that neoplastic transformation of one stem cell or glial precursor cell results in glioma initiation and development [25]. Overall, GCSCs and neural stem cells (NSC) share a number of similar characteristics, some of them also found in the accepted cancer stem-cell definition: extensive self-renewal ability and multipotency [25, 26]. Cancer initiation after *in vivo* injection, aberrant/blocked differentiation and genetic alterations are common features of both GCSCs and leukaemia stem cells. Importantly, the techniques used to discriminate cancer stem cells from the bulk of tumour cells and normal cells, such as xenograft transplantations and neurosphere cultures, frequently underestimate the frequency of cancer stem cells. Lately, evidence has been accumulating, showing that normal NSCs or neural progenitor cells (NPCs) can also initiate glioma, *via* activation of Notch signalling [27]. One underlying hypothesis is that transformation of NSCs or non-committed NPCs would generate high-grade gliomas [25], while low-grade gliomas are generated when lineage-committed progenitors (*e.g*. oligodendrocyte lineage-restricted progenitors, astrocyte lineage-restricted progenitor) are transformed [28]. Neural stem cells have been used as substrate for transformation to high-grade glioma when tumour suppressors, p16INK4a and p19ARF, were knocked out and active forms of epidermal growth factor receptor (EGFR) were introduced [29]. On the other hand, mature astrocytes can be transformed (and de-differentiated) when tumour suppressors, p16INK4a and p19ARF, were inactivated and cells maintained in serum-free conditions and EGF [29]. Thus, irrespective of the cell of origin, an NSC or a differentiated astrocyte, events that lead to loss of p16INK4a and p19ARF and constitutive signalling *via* EGFR, possibly leading to a loss of differentiation, eventually provoke highly malignant (high-grade) glioma. Epidermal growth factor was reported to induce GCSC renewal by promoting expression of the inhibitor of differentiation 3 (ID3), and subsequent ID3-induced cytokines IL-6 and IL-8 [30]. On the other hand, ID3 also suppresses invasiveness of GCSCs by inhibiting p27(KIP1)-RhoA that controls migration and matrix metalloproteinase expression [31]. Inhibition of EGFR will alleviate the differentiation block induced by ID3, but will promote invasiveness [32]. These reports illustrate the difficulties associated with treating these diseases with EGFR inhibitors.

A recent study using gene expression analysis further subdivided GBMs in several subtypes characterized by abnormalities in PDGFR-alpha, isocitrate dehydrogenase 1 (IDH1), EGFR and NF1 [33]. Consensus clustering of data from 202 samples and 1740 genes identified four subtypes with 210 gene signatures for each subtype, proneural, neural, classical and mesenchymal [33]. Interestingly, available treatment delays mortality in classical and mesenchymal subtypes only. These subtypes are close to the previously described molecular subclasses of high-grade glioma [34], although differences exist. Whether these GBM subgroups are associated with different cells of origin or with different mutations in the same initiating cell type remains to be established.

### Analogies with blood myeloid malignancies of different grades

**Fig. 1:**
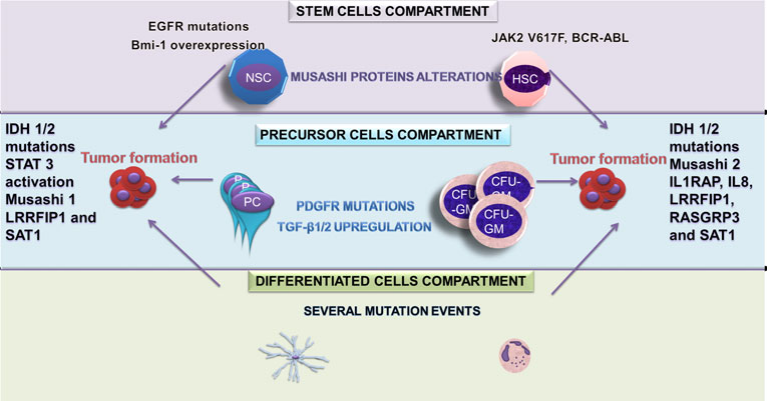
Schematic analogies between tumour initiation levels in neural and haematopoietic compartment. (**A**) Stem-cell compartment. Both neural stem cells (NSC) and haematopoietic stem cells (HSC) display Musashi protein alterations that may lead to tumour formation. Furthermore, NSC may express epidermal growth factor receptor mutations and/or Bmi-1 overexpression and HSC may acquire alterations in genes coding for different kinases, such as JAK2 (the V617F mutation) and ABL (the BCR-ABL translocation). (**B**) Precursor cell compartment. There are several tumour-initiating events, such as PDGFR mutations and transforming growth factor beta 1 and 2 up-regulation, that may lead to tumour formation in both neural precursor cell and haematopoietic precursor cells. It is postulated that such modified precursors acquire stem-cell properties, like self-renewal, and represent a tumour cell reservoir. (**C**) Differentiated cell compartment. Cancer stem cells can result from terminally differentiated cells, which acquire several genetic mutations in both glial and haematopoietic compartments, de-differentiate, become immortalized and perpetuate the malignant phenotype.

A parallel can be drawn with oncogenesis in the blood system (Fig. 1). Acute leukaemia and, more specifically, blast transformation of chronic leukaemia are thought to derive from rather committed progenitors (*i.e*. Colony Forming Unit Granulocyte Macrophage CFU-GM) that acquired self-renewal [35]. In contrast, chronic conditions such as human MPNs and myelodysplasia syndromes are because of transformation of an HSC or of an immediate HSC descendent with lympho-myeloid potential [36, 37]. Secondary acute myeloid leukaemia (AML) on MPNs or myelodysplastic syndromes are notoriously difficult to treat, and bone marrow transplantation attempts fail to be effective because such AMLs are resistant to treatment, with lack of complete remission. A major advance came with the description of several subsets of HSCs [38] such as myeloid-biased, lymphoid-biased, myeloid lymphoid-balanced and others [39–41], which exhibit different transplantation characteristics and predominance with age [41]. Pointing out the stated analogy, subsets of NSCs might as well be able to transform into different tumour types.

Transforming growth factor beta (TGF-β), which is also important for glioblastoma initiating cells, is able to stimulate self-renewal of myeloid-biased HSCs, and inhibit renewal of lymphoid-biased HSCs [41]. The major challenges are to actually elucidate the heterogeneity in leukaemia-initiating cells [42] and to somehow be able to isolate, study and target HSCs that carry mutations such as BCR-ABL, as they apparently behave quite similarly to their normal counterparts in several assays. Recently, the interleukin (IL) receptor-associated protein 1 (IRAP1) was detected specifically on BCR-ABL mutated, but not on normal HSCs [43], and also was detected in stem and progenitor cells of AML patients [44], thus opening the path to use it for specific detection of cancer-initiating cells and targeted treatment.

A number of transcripts were found enriched in stem cells of AML, such as IL1RAP, IL-8, LRRFIP1 (Leucine Rich Repeat in FLII or in Flightless homologue) interacting protein 1, miR-21, RASGRP3 and SAT1 (Spermine/Spermidine N1 Acetyl Transferase) [44]. Interestingly, LRRFIP1 and SAT1 are linked to the mesenchymal subtype of GBM, while another IL1RAP family member, IL1RAPL1, was linked to the proneural subtype [33]. However, SAM (Rank-ordered Significance Analysis of Microarrays) and ROC (Rank-ordered ROC lists for all pair wise comparisons) analyses suggested that IL-8 could be linked to the mesenchymal subtype, IL1RAP could be linked to both the classical and mesenchymal subtypes. This surprising commonality might suggest that the term ‘leukaemia of the brain’ might be appropriate for gliomas.

Furthermore, while in chronic myeloid cancers, such as BCR-ABL-negative MPNs, the main molecular basis is represented by mutations in Janus kinase 2 (JAK2) or cytokine receptors leading to constitutive JAK-signal transducer and activator of transcription (STAT) signalling [45], in secondary leukaemia and in the more severe MPN, many more mutations, especially in epigenetic regulators and genes coding for transcription factors, have been identified[6]. Furthermore, in myelodysplastic conditions, differentiation programmes are blocked, but proliferation of early committed progenitors is enhanced, while they also derive from a mutated or modified HSC [5].

A major sequencing effort directed to *de novo* acute myeloid leukaemia revealed several novel facts [46]. First, HSCs acquire a significant number of mutations before any driver leukaemia is acquired (∼5–10/year). They are usually silent functionally, but they accumulate, their nature is random and different from an individual to another and reflect the environment, unique exposures and polymorphisms in repair and other genes. Once driver mutations occur for leukaemia, then all these previous mutations are captured and carried by the clone as it expands. While only one or two additional mutations are required after the first driver, signalling in these clones depends on the other previous mutations as well, as they cooperate with the driver mutations [46]. It remains to be determined whether stem cells in the CNS might also acquire mutations over the years, and whether known drivers for glioma cooperate with those to induce progression to high-grade gliomas.

### Cancer stem cells in tumours with different grades of malignancy

Glioblastoma cells have the ability to form neurospheres [47]. The number of *in vitro* isolated neurospheres directly correlates with the growth rate and invasive pattern of the tumours formed when injected into immune-compromised mice. In contrast to neurospheres isolated from normal adult tissue, neurospheres isolated from human tumours contain genetic alterations and undergo aberrant proliferation and differentiation [48–51].

The study of cancer stem cells involves the following general work-flow: (*i*) stem-cell isolation from relevant tissues using specific markers, (*ii*) development of neurospheres, (*iii*) *in vitro* differentiation of neurospheres into neurons, astrocytes and oligodendrocytes [47, 52] and (*iv*) tumour formation when injecting tumour-derived neurospheres into immune-compromised mice.

The general present consensus is that the GCSCs share a number of characteristics with normal NSCs, namely self-renewal, pluripotency and neurosphere formation, while they display heterogeneity when injected into immune-compromised mice [20].

When analysing GCSCs in comparison with NSCs, the former are more resistant to treatments, are more prone to develop new tumours and to give rise to recurrence [20]. Differences probably exist between GCSCs and NSCs in terms of proliferation rate and subsequent various markers [20]. Extrapolating from another type of tumour, medulloblastoma, in humans, 30% of nestin-expressing medulloblastoma stem cells in the perivascular niche are proliferating, as compared to less than 1% of NSCs in subventricular zone (SVZ) [53]. Epigenetic mechanisms were invoked to explain the phenotype of NSCs, similarly to that of HSCs or other quiescent tissue stem cells, but exactly which signal maintains NSC quiescence remains to be determined. In the haematopoietic system, it has recently been demonstrated that the cytokine thrombopoietin (Tpo), which activates JAK2, STAT3 and STAT5, is responsible in the bone marrow niches for maintaining the quiescence of HSCs [54, 55]. In diseases where platelet number increases (thrombocytosis), levels of Tpo in plasma are very low because platelets are responsible for Tpo clearance. In these situations, HSCs' anomalies are observed with increased HSC cycling and exhaustion [56]. In contrast, Tpo strongly stimulates proliferation of committed myeloid progenitors [57]. It is tempting to speculate that in an analogous manner, a cytokine, possibly a member of the IL-6 type (also strong activator of JAK-STAT signalling, especially STAT3), might promote NSC quiescence, while stimulating the expansion of committed progenitors. A defect in the signalling response downstream of such receptor could switch the cytokine action from promoting quiescence to promoting renewal.

It is generally considered that cancer stem cells might acquire mutations that propel immortality, self-renewal and variable differentiation blocks in their progenitors [20]. It is also possible that they are much more heterogeneous than NSCs, given that several clonal cancer stem cells might be independently generated on the background of one founding clone; the mechanisms controlling this process remained elusive [13, 58, 59].

Regarding malignant *versus* benign tumour-initiating cells, several markers were suggested to discriminate between high- and low-grade gliomas. Although high-grade and low-grade gliomas share the expression pattern of glial progenitor cell surface markers, only high-grade gliomas show neuronal differentiation potential [28].

It remains to be established whether the *in vivo* multi-lineage differentiation capacity observed in high-grade tumours can be gained following progression from low-grade to high-grade gliomas. Most likely, low-grade gliomas are arrested at more advanced differentiation stages. Several markers (see below) were identified in relation to malignant and benign intracranial tumours, markers that characterize the stem cells contained by these tumours.

Studies that have compared benign astrocytoma, low-grade glioma and high-grade glioma (glioblastoma multiforme) have highlighted potentially important genetic events associated with glioma progression, such as methylation of DNA methylase 1 (DNAMT1), methyl guanine methyl transferase (MGMT) and EGFR promoters, chromosomal losses and gains, IDH1 mutations in low-grade astrocytoma [32, 60–62].

The therapy of brain tumours stumbled on the resistance supposed to reside in GCSCs, which are considered as true cancer reservoirs. Thus, even if most of the cells in a tumour can be destroyed, cancer stem cells may survive and regenerate the tumour [63, 64]. This is similar to the situation in blood and other cancers. Similar to cancer stem cells isolated from glioblastomas, stem cells of low-grade gliomas [28, 65–67] are more resistant to chemotherapeutics than their differentiated daughter cells [21].

### Cancer stem-cell microenvironment – relationship with the niche

The self-renewing and differentiation potential of stem cells results from their integration of cell-autonomous properties and ‘stromal’ extrinsic signals, generated by the niche [1].

**Fig. 2:**
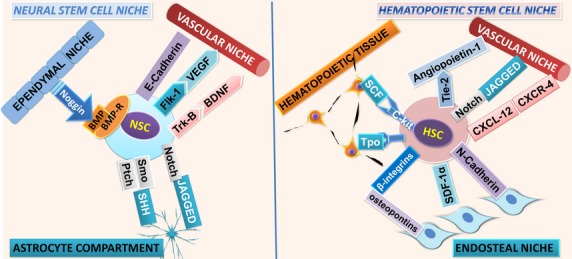
Molecular mediators involved in adult stem-cell renewal within neural and haematopoietic stem-cell (HSC) niche. Both neural and HSCs communicate with their niches through similar signalling pathways, such as Notch. Some factors are responsible for homoeostasis of each particular niche, such as Tpo for the haematopoietic system, which is required for HSC's quiescence and maintenance of HSC reservoir and brain-derived nerve factor (BDNF) for neural stem cells (NSCs). It is not known whether certain mutations acquired by HSC or NSC are positively selected by defects in the niche, which apply a certain pressure of selection on stem and progenitor cells. Figure adapted from [68] and [75].

The stem-cell niche (SCN) represents not only ‘an anchoring site’ for stem cells, which appears to communicate and ‘bind’ to ‘docking’ cells by gap and tight junctions, but it also represent a site for generation of extrinsic factors that control stem-cell number, proliferation, division, migration close to capillaries and probably support their stemness [57]. Several SCNs have been described in mammals at haematopoietic, intestinal, epidermal and neural levels. They all share the common feature of vascular niche–stem cell cooperation (Fig. 2). In addition, HSC exchanges signals with the osteoblasts that form the endosteal niche, *via* cell–matrix interactions, N-cadherin-mediated cellular contacts [68] and diffusible factors [69]. Extensive studies exist on the endosteal and vascular niches for HSCs in the bone marrow, and intense research is ongoing on the very important role of the niche in blood formation. Defects in the niche have recently been shown to induce in humans dysplastic features and neoplastic transformation [70]. Furthermore, a significant fraction of GBM endothelial cells appeared to have derived from the neoplastic clone [70], suggesting that tumours create their own niches. Transdifferentiation of glioblastoma cells into vascular endothelia cells was reported [71]. A similar type of observation has been made in human MPNs where endothelial cells in certain vessels were found to carry the JAK2 V617F mutation, especially in patients with Budd-Chiari syndrome and portal vein thrombosis and thus to be derived from the MPN stem cell [72].

Neural stem cells have been shown to be embedded within a vascular microenvironment [53], CD133^+^, nestin+ tumour cells (containing the GCSCs) being located next to capillaries in brain tumours [1].

Two SCNs have been recognized in the mammalian brain, one in the SVZ and the other in the subgranular zone. Neural stem cells isolated from the SVZ differentiate *in vitro* into both neurons and glia [73, 74] and are regulated by contact-dependent factors and diffusible signals (Fig. 2) [75].

Direct physical interactions between stem cells and the non-stem cells are a very important part of the niche dynamics. E-Cadherin is expressed by NSCs and regulates NSC self-renewal in the murine forebrain [76]. In human brain, ribbons of astrocytes were identified in the SVZ lining the lateral ventricles [77]. Neurospheres displaying tri-lineage differentiation could be derived from single cells contained in this region.

Experiments on cancer stem cells have indicated that SCNs play major roles in the regulation of stem-cell behaviour, further emphasizing their importance in controlling self-renewal rate, proliferation, cell-lineage, and as such, can contribute to cancer development and to the degree of oncogenic transformation [78, 79]. Using a mouse model of medulloblastoma, Hambardzumyan *et al*. show that, while the main brain tumour population is affected and undergoes apoptosis upon irradiation, tumour cells in the perivascular niche are relatively resistant to radiation and when overcoming irradiation, they show substantial activation of the Akt/mTOR pathway [53].

## Markers associated with tumour-initiating cells

### CD133 as cancer stem-cell marker

A stem-cell marker now extensively used as a surface marker to identify and isolate cancer stem cells in malignant brain tumours, CD133 (a transmembrane protein also called prominin 1), has been subjected lately to much controversy [5]. CD133 was the first identified member of the prominin family of pentaspan membrane proteins [80] in the form of a single-chain polypeptide of 865 amino acids (MW = 120 kD). There have been identified 12 different prominin-1 splice variants, having as a main feature the rapid down-regulation during cell differentiation [81, 82].

CD133 served as a marker for *in vitro* clonogenicity [50] and also for the self-renewal characteristic of GCSCs. CD133 is rapidly down-regulated during cell differentiation, this characteristic being used to identify and isolate stem cells and progenitor cells in several tissues [82].

One favourite hypothesis was that gliomas arise from cancer stem cells that are CD133-positive, although some gliomas contain CD133-negative GCSCs [2, 50, 52, 63, 83, 84]. There is a wide-range of variation in CD133-positive percentages (0.1–50% in GBM patients) [2, 52, 85], this variability being further discussed. Lineage genetic analysis of CD133-positive precursors displayed a 24-gene signature profile that allowed classification in type I proneural GCSCs (resembling foetal NSC), whereas type II GCSCs exhibit a mesenchymal gene expression profile resembling adult NSCs [86].

Glioblastoma multiforme are heterogeneous with respect to CD133 expression and certain CD133-low or -negative GBM exhibit highly malignant features and invasive growth [87]. Gene expression studies comparing proneuronal with mesenchymal GBM subtypes showed, nevertheless, that expression of CD133 was enhanced in mesenchymal GBM and in proneuronal GBM that recurred and changed upon recurrence into a more mesenchymal type [34].

Neither the expression of stemness genes nor the long-term self-renewal capacities were different between CD133-positive and CD133-negative cells in gliomas [88]. In every GBM patient, multiple kinds of tumour-initiating cells, such as CD133-negative and CD133-positive cells, can co-exist [87]. However, the proportion of CD133-positive cells can be a possible prognostic factor for adverse progression-free survival and overall survival independent of tumour grade, extent of resection or patient age. The proportion of CD133-positive cells was an independent risk factor for tumour re-growth and time to malignant progression in WHO grade II and grade III tumours [89]. High levels of CD133 expression may be associated with a high risk of dissemination, although low CD133 expression may not exclude this possibility [14].

CD133-positive GCSCs are already recognized to be markedly resistant to conventional anticancer therapies [63, 64]. A homeobox gene expression pattern signature (which associates with CD133 expression) can predict the poor survival in treated patients. This has been the first clinical evidence of a ‘glioma stem cell’ or ‘self-renewal’ phenotype in treatment resistance of GBM [90].

Gene expression pattern of GCSCs can change depending on *in vivo* and *in vitro* conditions, without altering their differentiation property. Therefore, CD133-positive and CD133-negative GBM cells grown *in vitro* in particular conditions can differentiate into various brain cells that express GFAP (astrocyte), Tuj1 (neuron), or O4 (oligodendrocyte) [87]. Furthermore, in xenograft transplantation studies, CD133-negative cells could generate CD133-positive cells upon tumourigenesis [91].

Combinations of various standard markers have recently been studied. The presence of double CD133^+^/Ki67^+^ positive cells can constitute a prognostic factor of disease progression and poor clinical outcome [92]. Increased expression detected by MIB-1 monoclonal antibody has been associated with shorter time interval before development of dissemination in patients with disseminating GBM [93].

The tumour microenvironment, which is known to favour a hypoxic milieu, promotes the self-renewal capacity of CD133-positive human GCSCs [94]. CD133-positive cells present an up-regulated HIF-1alpha and also express chemokine receptor CXCR4 (CD184), cell surface glycoprotein CD44^low^ and A2B5 surface marker for neuronal and glial cells. Recently, it was demonstrated that CXCR4 expressed by bone marrow-derived neural progenitor/stem cells is necessary for these cells to move and invade the extracellular matrix following a gradient of glioma soluble factors [95].

Although CD133 positivity was correlated with poor clinical prognosis in brain tumours, the use of CD133 as a unique glioma stem-cell marker is not sufficient to mark the entire self-renewing tumour cell reservoir [88]. CD133-positive brain tumour cells could become therapy targets to eradicate brain tumours because CD133-positive glioma stem cells could be a potential source for infiltration of surrounding tissue [5].

### Hand-in-hand CD133 and nestin expression

Another marker studied in close relation to GCSCs is nestin, involved in several important cellular functions, such as signalling, cytoskeleton organization and metabolism. It is associated with the characteristics of multi-lineage progenitor cells, like proliferation and migration, and is down-regulated upon differentiation [87, 96]. Down-regulated nestin may be re-expressed in the adult organism under neoplastic transformation [96]. Nestin is expressed more frequently in higher grade gliomas, being predictive of a significantly lower 5-year survival rate [97]. The level of nestin expression in tumour cells was found to be higher in primary tumours from patients with dissemination, when compared with patients without dissemination [14].

Various recent reports have been published stating the ‘hand-in-hand’ relationship of these two markers. Nestin and CD133 were reported in a variety of brain tumours [98]. The correlation of nestin and CD133 expression has recently been thought to mark the grading, and its clinical outcome predictive power was being tested [96]. Nestin+/CD133^+^ patients had the poorest prognosis. Recent data show that the co-expression of CD133 and nestin was found around CD31^+^ blood vessels. The combination of CD133/CD31 or nestin/CD31 was found in both endothelial cells and cancer stem cells [97].

Overall nestin/CD133 positivity was associated with a poor prognosis and better correlated with clinical course and survival than the histological grading. Therefore, their combination can be a potential indicator of the aggressiveness of gliomas and to be a possible marker of tumour burden and recurrence [65, 67, 96, 99]. The transcriptional/chromatin/epigenetic bases for this co-ordinated induction of nestin and CD133 need to be determined.

### Musashi-1 anti-differentiation regulator

Musashi proteins are considered regulators of translation and cell fate. The expression of Musashi-1 was demonstrated in GBM stem cells (Table 1) and in neurosphere cultures established from surgical specimens [100]. The expression of Musashi-1 has been consistently correlated with both the grade of the malignancy and proliferative activity in gliomas [101] and in neurospheres derived from brain tumours [48], thus suggesting that this molecule may mark proliferating GCSCs. These cells express stem-cell markers, besides Musashi-1, such as Sox2, nestin, maternal embryonic leucine zipper kinase (MELK) and CD133 [48, 52, 84, 102], and were shown to be able to re-initiate tumourigenesis. Neurospheres treated with TGF-β or leukaemia inhibitory factor (LIF) maintained the expression of neuroprogenitor markers (Musashi-1, Sox2, and nestin) and an undifferentiated cellular phenotype [103].

**Table 1:** Key molecules operating in normal neural stem cells and in glioma cancer stem cells

Abbreviation/Name	Function	Roles	Involvement in tumourigenesis	Ref.
**Bmi-1** Polycomb complex protein	Transcription factor (chromatin regulator) gene transcription repressor	Proliferation of neural and haematopoietic stem cells, but not involved in progenitors functionality. Regulated by Shh; regulates p16/INK	Required for cancer stem-cell function. Overexpressed in brain tumours	[181]
**IDH1/IDH2** Isocitrate dehydrogenase 1/2	Metabolic enzyme	Catalyses neomorphic formation of 2-hydroxyglutarate	Frequent in astrocytomas, oligodendrogliomas and glioblastomas; IDH1/2 mutations in acute myeloid leukaemia correlate with better outcome	[183, 185]
**TET2** Tet methylcytosine dioxygenase 2	Enzyme	Catalyses the conversion of the modified DNA base methylcytosine into 5-hydroxymethylcytosine	Frequently mutated in myeloproliferative disorders and brain cancers; absence of TET2 expression is exclusive with IDH1/2 mutations	[149]
**Notch1** Neurogenic locus notch homologue protein 1	Membrane receptor for Jagged1, Jagged2, Delta1	Involved in the maturation of NSC and NSC function's maintenance	Collaborates with epigenetic silencers to promote malignant tumours by Rb silencing	[191]
SOX-2 Transcription factor	Transcription factor	Involved in the proliferation and functionality of NSC and precursors	Expressed in malignant gliomas (gene expression profiling), and paediatric brain tumours	[107, 192]
**Shh** Sonic hedgehog protein	Signalling protein	Proliferation of NSC and GSC	Activation of Shh pathway in brainstem glioma	[193]
**Ptch** Protein patched homologue 1	Shh receptor tumour suppressor function	Proliferation of NSC and GSC	Ptch highly expressed in astrocytoma, oligodendroglioma, GBM	[154]
**Gli1, Gli2, Gli3**	Components of Shh pathway (transcription factors, activators or repressors)	Components of Shh and Wnt pathways. Gli-1 protein expressed in NSC	Originally isolated from glioblastoma. GLI expressed in GBM, astrocytoma, oligodendroglioma	[154]
**N-myc** Proto-oncogene protein	Transcription factor	Neural progenitor cells expansion and inhibition of neuronal differentiation	N-MYC gene is amplified in some human brain tumours. overexpression correlates with tumour progression	[194]
**p53** Cellular tumour antigen p53	Transcription regulator	Negative regulation of NSC proliferation, tumour suppressor	Synergistic with Ptch, Rb, PARP-1, Ink4c. Mutated in some brain tumours	[195]
**Beta-catenin** Catenin beta-1	Cell adhesion and signal transduction; transcription factor (Wnt pathway)	Important for NSC/progenitor cell proliferation	Mutated in some human glioma, medulloblastomas	[196]
**MSI1, Musashi,** RNA-binding protein Musashi homologue 1	Regulates the expression of target mRNAs at the translation level	Protein alteration favours tumourigenesis	NSC and HSC display Musashi alterations. Consistently correlated with the tumour proliferation in gliomas	[101]
**PTEN** Phosphatase and tensin homologue	Tumour suppressor	Inhibitor of NSC proliferation	Mutated in human glioblastoma	[93]
**PROM1/CD133** Prominin-1	Membrane protein Stem-cell marker	Maintenance of stem-cell properties (differentiation suppressor)	Lost during CSC differentiation, different glycosylation pattern in CSC	[5]
**EGFR** Epidermal growth factor receptor	Membrane receptor; tyrosine kinase	EGF-dependent NSC proliferation	Often amplified and mutated in high-grade gliomas	[90, 197]
**PDGFRA** Platelet-derived growth factor receptor-alpha protein	Membrane receptor; tyrosine kinase	Conversion of oligodendrocyte progenitors into neural stem-like cells	Expressed in gliomas. Amplified and mutated in glioblastoma	[102]
**Nestin**	Intermediate filament	Trafficking and distribution of factors during progenitor cell division	Expression correlated with tumour grade	[96]
**MELK** Maternal embryonic leucine zipper kinase	Protein kinase	Functional regulation of transcription factor	Highly expressed in NSC and brain tumours	[112, 113]
**p21 cip1** Cyclin-dependent kinase inhibitor 1	Kinase inhibitor	Quiescence of NSC and HSC. Mediates proliferation inhibition by p53 pathway	Down-regulated in tumour transformation	[198]
**p16-INK4a/CDKN2A** Cyclin-dependent kinase inhibitor 2A	p53 activator	Inhibitor of NSC proliferation; over-expressed in Bmi−/− mutant stem cells	Absent or altered expression in various GBM	[199]

Interestingly, it has been shown that the blast crisis (AML transformation) of CML involves another member of the Musashi family, namely Musashi-2, which represses the protein Numb [104]. High levels of Musashi 2 are predictive of blast transformation and poor prognosis [104]. In GCSCs cells, Y box-protein 1 (YB-1), SOX2, Musashi-1, Bmi-1 and nestin are co-ordinately expressed [105]. Interestingly, YB-1 marks both NSCs and GCSCs of gliomas and is required for normal brain formation [34]. In glioma cells, the reduction of Musashi-1 induced the prolongation of the cell cycle, while Notch and PI3 kinase-Akt signalling was reduced, *via* up-regulation of Numb and phosphatase and tensin homologue (PTEN), respectively [106].

The above-mentioned studies and others suggest that Musashi proteins play a major role in blocking differentiation, which is a pre-requisite for high levels of malignancy by promoting the activation of major signalling pathways. This again is a theme well known from leukaemias and myelodysplastic disorders.

### Other markers

One of the main genes involved in the self-renewal of several stem cells, in particular NSCs, is SOX2 [107]. SOX2 plays a major role in the recently discovered reprogramming capacity of the four transcription factors SOX2, Klf4, Oct4 and Myc (known as reprogramming factors) [108]. It was recently shown that once SOX2 is induced, reprogramming occurs *via* a hierarchical programme [109]. SOX2 expression in glioma stem cells was recently demonstrated [100]; it strongly correlates with the maintenance of stemness [103] and it is regulated by the TGF-β signalling pathway [110]. Therefore, SOX2 and its downstream effectors would be targets for glioblastoma therapy [111]. The loss of proliferation ability and tumourigenicity of glioblastoma stem cells was recently demonstrated *in vivo* after SOX2 silencing [111].

Maternal embryonic leucine zipper kinase, one of the AMP/snf1 kinases is highly expressed in NSC and malignant brain tumours [112]. Its expression was found increased in brain tumour stem cell-enriched cell cultures. Animal models, using transgenic MELK-reporter mice, indicated that MELK is expressed in NSCs. Recent *in vitro* studies demonstrated that MELK is required for NSC self-renewal, for proliferation of putative GCSCs. The expression of MELK in GBM stem cells was demonstrated by RT-PCR. The loss of MELK expression during differentiation was reduced in CD133-negative cells compared with CD133-positive cells [5, 113]. Interestingly, the level of expression of MELK correlates with the malignancy grade in human astrocytomas: MELK was found to be highly expressed in highly invasive glioblastoma multiforme as opposed to the benign pilocytic astrocytoma [112]. In a recent study, the expression of MELK was found up-regulated in glioblastoma tissue [114]. Therefore, MELK could be used as a therapeutic target. Relevant markers and signalling molecules involved in GCSCs in glioblastoma are summarized in Table 1. A parallel is drawn with respect to HSCs and stem cell-initiating blood cancers.

## Signalling pathway deregulations

As already stated, GCSCs share properties with NSCs, but probably subtle differences reside at both genetic and epigenetic levels, which lead to immortal growth, a block in differentiation and invasiveness. In brain tumours, elements of signalling pathways operating in stem and/or stem-like cells could be regarded either as potential markers, or as therapeutic targets, or *ex vivo*, to identify or isolate different cell types [115].

The description of both normal signalling pathways and clinical features of benign or malignant brain tumours is beyond the purpose of the present section. We provide here only a short overview of recent data linking elements of signalling networks with the concepts of ‘marker’ or ‘therapeutic target’, especially in GSCSs. However, it is important to recall that high-grade gliomas are highly resistant to treatment and that novel approaches are necessary, whereby several deregulated pathways need to be targeted simultaneously.

### Receptor tyrosine kinases, downstream PI-3′-kinase (PI3K) and Akt

Epidermal growth factor (EGF) and Fibroblast growth factor (FGF) support the growth of both NSCs and GCSCs *in vitro*. In general, tyrosine kinase receptors (EGFR, PDGFR, FGFR, Met-receptor) have been much studied in gliomas [51]. The downstream PI3K/Akt/mTOR pathway is one of the most important and best characterized pathways in gliomas. Malignant gliomas, particularly GBM, frequently display amplification of EGFR, specific activating mutations in EGFR and, to a lesser extent PDGFR amplification [33]. One consequence of tyrosine kinase receptor signalling activation is represented by constitutive activation of the PI3K pathway, which is critical for cell survival and several cancers, notably blood malignancies [116]. Ak mouse breed thymoma is a major downstream component of the PI3K pathway, critical to the survival and growth of gliomas [51]. In these tumours, both the activity of the Akt pathway and the expression of stem-cell markers correlate with aggressive behaviour and resistance. In human gliomas, Akt signalling correlates with poor prognosis in patients. Several studies using *in vitro* models for gliomas have proven that Akt contributes to tumour formation and growth [20]. The role of Akt is complemented by low constitutive mitogen activated protein (MAP)-kinase Erk activation that correlates with cell proliferation, the authors pointing out their importance as future therapy targets [117]. It is interesting that in cells expressing certain EGFR mutants, levels of Erk1,2 MAP-kinase activity are quite low, presumably because of induction of MAP-kinase phosphatases [118], which would prevent excessive MAP-kinase activation, which could have differentiation or anti-proliferative effects. What seems specific for gliomas is activation of the mTOR pathway (mammalian target of rapamycin) by EGF *via* protein kinase C alpha, apparently through an independent circuit from the classical Akt-mTOR activation [119]. Interestingly, ectodomain cleavage of EGF family members *via* a disintegrin and metallo-proteinases (ADAMs), which contributes to generation of proliferative signals, involves activation of protein kinase C alpha. This leads to phosphorylation of Ser/Thr residues in cytosolic domains of transmembrane EGF precursors, which promotes selection of these proteins as substrates for ADAMs [120]. These results would suggest that protein kinase C inhibitors would be candidates for combination therapies.

Based on results obtained in other cancers, pan type I PI3K inhibitors (covering the alpha, beta, delta and gamma p110 isoforms) will likely be effective [116], in contrast to isoform-specific inhibitors. In GBMs, inhibitors of PI3K alone appear to block proliferation, but not induce apoptosis [121] and so, new combinations are being tested, such as the PI3K inhibitor PIK-90 and the cyclin-dependent kinase CDK 1/2 inhibitor (roscovitine), which showed synthetic lethality, defined as induction of glioma cell death only by the combination and not by single molecule treatment [122].

This nicely parallels the situation in chronic myeloid cancers, such as myelofibrosis where recently two independent reports showed that in myelofibrosis and in MPN models, the combination of JAK2 inhibitors and PI3K (pan type I) inhibitors, such as ZSTK424, GDC0941 and NVP-BEZ235 (also an mTOR inhibitor), synergize to prevent cytokine-independent proliferation, which is characteristic of MPNs [123, 124]. Furthermore, in a phase II clinical trial, an mTOR inhibitor, Everolimus, showed efficacy [125].

Not only EGFR gene amplification is the most common alteration in high-grade glioma, but also 50% of the EGFR amplified tumours express a constitutively active form of EGFR, which is required for glioma growth [126]. A paracrine mechanism driven by Delta-EGFR, with secretion of IL-6 and LIF was shown to recruit wild-type EGFR-expressing glioma cells into accelerated *in vivo* proliferation [126]. Furthermore, one pathway of resistance to EGFR inhibitors in EGFR over-expressing/Delta-EGFR glioblastomas is represented by phosphorylation of PTEN at tyrosine 240 [127]. As mentioned above, EGF signalling cooperates with inhibition of tumour suppressors in inducing inhibitors of differentiation and ultimately inflammatory cytokines (IL-6 and IL-8) [29, 30].

Epidermal growth factor receptor is overexpressed and/or mutated in many carcinomas, including lung, breast, colon, head and neck, prostate and ovarian, but not in haematological disorders. Nevertheless, some mutations are specific for glioblastoma [128]. Oncogenic EGFR signalling networks are being described in glioma and were shown to be both robust and plastic, able to adapt to single molecule inhibition, and these are becoming targets for combination of EGFR and downstream pathway inhibitors [21].

The genetic alterations found throughout the tumour bulk in gliomas, such as loss of p53 and PTEN, result in significant resistance to therapy in all cell types. PTEN and p53 are shown to negatively regulate NSC self-renewal, and their deficiency leads to a significant decrease in the level of apoptosis in neurospheres [20]. Again, the parallel with HSCs is that p53 was recently shown to control HSC quiescence [129]. The inhibition of Akt with pharmacological inhibitors disrupts neurosphere formation, induces apoptosis, reduces migration and invasion *in vitro* and significantly delays intracranial tumour formation [79, 115].

So far, clinical trials (http://www.clinicaltrials.gov) using tyrosine kinase inhibitors have failed to delay the development of glioblastoma, by contrast to leukaemia. The precise reasons for failure are unknown, but pathological RTK signalling might be a late event promoting survival and proliferation in glioma, while the initiating event remains unknown. In contrast, in chronic myeloid leukemia, the initiating event is represented by BCR-ABL, while in several other myeloid cancers the initiating events are PDGFR translocations, so therapy in those cases would target in the initiating events. Furthermore, high-grade gliomas resemble more acute leukaemias, especially secondary AML on MPN or MDS, and in those conditions kinase inhibitors are also inefficient. The PI3K pathway is constitutively active in AML, either by overexpression, namely of the p110 PI3K delta, other catalytic subunits or activating mutations [130–132].

### PDGF

Glioblastoma cells express all PDGF ligands and their specific receptors, PDGFRA (alpha) and B (beta). This abundant expression leads to the stimulation of the glial tumour cells by the tandem PDGF/PDGFR, especially PDGFRA. Overexpression of PDGFRA is present in human low-grade gliomas and further enhanced by gene amplification in a subset of high-grade glioblastoma, whereas PDGF-B induces angiogenesis characteristic for secondary glioblastoma [133]. Importantly, intra-cerebral injection of a PDGF-encoding retrovirus can induce brain tumours of oligo- or monoclonal origin, which co-express PDGF B-chain and alpha-receptor (PDGFRA) mRNA [134]. Most of these PDGF-induced tumours display characteristics of glioblastoma multiforme or of a primitive neuro-ectodermal tumour, and are nestin-positive, suggesting their derivation from an immature neuroglial progenitor [134]. Furthermore, NSCs of adult brain SVZ were shown to express PDGFRA, which is physiologically required for oligodendrogenesis; these cells respond to PDGF treatment by a block in neurogenesis and hyperplasia resembling early stages of glioma oncogenesis [102].

Imatinib, an inhibitor of PDGFR alpha and beta kinases, as well as other selected tyrosine kinases (Abl and KIT), is indicated for treatment of CML, myeloid cancers with PDGFR translocations and gastrointestinal stromal tumour. Unfortunately, imatinib, although well tolerated in association with hydroxyurea, did not show clinically meaningful anti-tumour activity in a phase II clinical trial with recurrent GBM [135]. This combination is now completing a phase III clinical trial for patients with temozolomide-resistant progressive glioblastoma (http://www.clinicaltrials.gov/ct2/show/study/NCT00154375?term=temozolomide±AND±imatinib&rank=2).

The expression of PDGFRA protein and detection of its phosphorylation were assessed in the randomized trial CSTI571BDE40 comparing GBMs randomized for treatment with imatinib and hydroxyurea *versus* hydroxyurea alone. In this trial, it was determined that PDGFRA expression and phosphorylation exert significant prognostic value for shorter survival, but did not confer responsiveness to imatinib therapy [136]. In contrast, imatinib is quite effective in haematopoietic malignancies associated with translocations or mutations in PDGFR [137], while hydroxyurea is a standard treatment for MPNs.

### Notch

In the CNS, Notch signalling pathway prevents nestin degradation during stem-cell differentiation, through a mechanism that possibly involves ubiquitin-proteasome pathways [138]. Constitutive Notch signalling in NSCs is tumourigenic and promotes astroglial lineage entry [139].

Notch ligands, receptors and targets have been found in GCSCs to play an important role in the pathogenesis of brain tumours. These tumours, which contain stem-like cancer cells, exhibit higher Notch activity [28]. Increased Notch activity promotes tumour growth, whereas Notch pathway blockade inhibits proliferation and/or survival. Knockdown of Notch-1 and its ligands induces apoptosis and inhibits proliferation [140]. Notch signalling can directly up-regulate nestin expression in gliomas. In addition, a constitutive activation of Notch signalling in glioma cell lines promotes growth and increases the formation of neurosphere-like colonies [138, 141].

Notch pathway seems to be intimately coupled to MAP-kinase signalling with proven crosstalk between the Notch pathway and EGFR signalling, which has been revealed in tumour angiogenesis [142]. Notch receptor expression can be correlated with receptors involved in angiogenesis like VEGFR2, VEGFR3 and PDGFRβ [114]. Notch signalling, and especially Delta-like 4, is involved in anti-VEGF therapy resistance [143, 144]. Overall, the inhibition of Notch signalling might therefore be a promising therapeutic avenue.

### TGF-β

Cancer initiating cells for GBM appear to be characterized by a CD44^high^/Id1^high^ phenotype [145]. These cells are stimulated by TGF-β and this contributes to oncogenicity [110], in accord with higher plasma levels of TGF-β in plasma of patients with glioblastoma [146]. Interestingly, in the haematopoietic system, it was shown that ‘myeloid-biased’ stem cells are stimulated by TGF-β. Those HSCs are usually the ones that acquire oncogenic mutations [41]. Massive parallel sequencing has shown that TGF-β1 and SOX4 are up-regulated in glioblastoma [145]. The cancer-initiating cells in glioblastoma were shown to be promoted to self-renew by TGF-β *via* the induction of LIF [103], a cytokine of the IL-6 family, which induces self-renewal of embryonic stem cells *via* activation of STAT3. Another cytokine, IL-10, correlated at both gene and protein levels with TGF-β2 expression. Both markers are overexpressed in tumour spheres formed by stem cell-derived cells (TSCs) in comparison with the primary cultured glioma cells (PCGCs), even if the origin was the same tumour. This overexpression in TSCs correlated with the pathological grade of the glioma [147]. Furthermore, high TGF-β signalling *via* Smad proteins is considered of poor prognosis in glioblastoma and can induce proliferation, provided that the PDGF-B gene can be induced by demethylation [148].

Of great therapeutic interest, inhibitors of TGF-β receptors target cancer stem cells located in perivascular niches [145]. On a molecular level, TGF-β stimulation appears to maintain the expression of transcription factors ID1 and ID3 (inhibitors of DNA-binding proteins), which further exert a negative effect on the DNA-binding capacity of basic helix-loop-helix transcription factors. How these ID proteins maintain the cancer stem-cell phenotype is not known, but the response of GCSCs to TGF-β receptor inhibitors by reducing ID1, ID3 proteins and cell surface CD44 is very important as such markers appear to be functionally linked to the cancer-initiating phenotype of GCSCs [145]. On the other hand, ID3 expression was suggested to be a mediator of the oncogenic effects of EGF signalling [149]; one way to reconcile these data would be to postulate some effects of ID3 that would be pro-oncogenic and others anti-oncogenic, and that inhibitors of ID3 induced when TGF-β receptors are inhibited would act on the oncogenic pathway.

### Bone morphogenetic proteins

Bone morphogenetic proteins (BMP) belong to the TGF superfamily, and were demonstrated to be involved in the control of cell growth, differentiation and maintenance of normal tissue and tumours. Bone morphogenetic proteins and especially BMP4 exert a negative action of GCSCs [150]. In xenotransplantation experiments, administration of BMP4 prevents tumour growth and mortality. The mechanism involved activation of BMP receptors, Smad activation and an anti-proliferative effect along with induction of neural differentiation [150]. Interestingly, BMPs are crucial for differentiation in CNS [151]. Both BMP type IA (BMPR-1A) and the type IB (BMPR-1B) receptors were detected in human glioma cells. In tumour cells, a significant increase in BMPR-IB was observed to be progressively expressed in malignant glioma compared with low-grade astrocytomas [152]. Epigenetic silencing of BMPR1B in a subset of gliomas leads to astroglial differentiation block [38]. Overall, mimicking events induced by BMP4 and identifying genes induced and repressed in this condition might lead to new targets of therapy.

### Sonic Hedgehog and Wnt

Sonic Hedgehog (SHH)-Gli signalling pathway regulates self-renewal and tumourigenic potential of GCSCs and active SHH signalling has been associated with glioblastoma [115, 153]. Inhibition of Hedgehog-Gli signalling suppresses self-renewal and proliferation, while increasing apoptosis [154, 155]. Gli activity was correlated with malignancy grade in PDGF-induced gliomas [53]. When the roles of Notch, Hedgehog and Wnt signalling were examined in xenotransplantation studies, Hedgehog was found to be indispensable for tumour formation [156].

Wnt-signalling activates the translocation of beta-catenin to the nucleus, where it acts as a transcription factor of specific target genes. Wnt-β-catenin signalling has proven roles in both normal stem cells and GCSCs [157]. Wnt-β-catenin signalling can contribute to radio-resistance in GCSCs and, probably, Wnt could be a therapeutic target for GCSCs in brain tumours [158], as this pathway is supporting motility/invasiveness of gliomas and is driving changes resembling epithelial to mesenchymal transition [159].

### STAT3 and STAT5

The family of STATs is highly involved in brain tumourigenesis. The human genome codes for seven STATs (STAT1, 2, 3, 4, 5A, 5B and 6), which are tyrosine phosphorylated by four JAKs linked to more than 30 cytokine receptors [45]. Upon cytokine binding to cytokine receptors, a conformational change usually occurs in receptors' transmembrane domains [160], which leads to activation of JAK, which are pre-bound to the cytosolic regions of receptors [161]. Janus kinases cross-phosphorylate each other and receptor cytosolic domains, which attract STAT proteins *via* their SH2 domains. Once bound to receptors, STATs become substrates of JAKs, become tyrosine phosphorylated in the transactivation domain, which leads to their dimerization to other STATs, detachment from receptor and migration in the nucleus where they regulate gene expression [162]. The link between the activation of STAT3 and glioblastoma biology has become increasingly convincing [163, 164]. STAT3 activation in conjunction with C/EBP-β correlates with mesenchymal transformation of glioblastomas and is inversely related to outcome [164, 165]. Inhibition of STAT3 with specific inhibitors disrupts proliferation and maintenance of GCSCs [166]. Several pathways upstream of STAT3 are active in GCSCs. Targeting these pathways inhibits STAT3 activation and GCSCs growth and self-renewal [167]. Thus, like other cancers, constitutive activation of STAT3 is likely to play an important role in the survival and proliferation of glioblastomas [26]. The identity of the tyrosine kinase responsible for constitutive STAT3 activation in glial cancers is unknown.

One gene that is highly expressed in highly invasive GBM in comparison with low-grade astrocytomas is IL-13 receptor alpha2 [112]. IL-13Ralpha2, initially discovered to be a ‘cancer testis’ or ‘tumour’ antigen, is also highly expressed at the surface of several types of cancer cells, especially glioblastomas [168, 169]. Its expression led to the suggestion of its use as a target for specific killing of cancer cells [170, 171]. The IL-13Ralpha2 has a 21% homology to IL-13Ralpha1, the *bona fide* receptor for IL-13 that forms a complex with IL-4Ralpha and mediates JAK and STAT6 activation in response to IL-13 [172]. IL-13Ralpha2 was recently demonstrated to have extraordinary high affinity for IL-13, thus explaining a decoy function for this receptor chain in eliminating circulating IL-13 [173]. This chain only possesses a 20-amino-acid cytosolic domain that cannot tether JAKs [172, 174]. But the receptor appears to mediate signalling *via* alternative pathways, leading to AP1 and TGF-β induction [175], and by promoting activation of STAT3 [176]. Interleukin-13Ralpha2 was also shown to activate MAP-kinase signalling in intestinal cells from inflammatory bowel disease patients [177]. It is therefore possible that signalling by highly expressed IL-13 receptor alpha2 is important for glioblastoma cells.

Constitutive STAT5 signalling has also been reported in GBM, and STAT5 has been linked in haematopoietic cells with anti-apoptotic signalling. Especially in patients who harbour EGFR overexpression and presence of the constitutively active delta-EGFR, STAT5b was shown to induce BclXL expression and survival of GBM cells [178].

In contrast, in haematopoietic cancers, especially human MPNs that do not harbour BCR-ABL or PDGFR translocations, the JAK2 V617F unique acquired mutation is prevalent, followed by mutations in Tpo receptor and negative regulators such as Cbl [45]. These mutations are associated with constitutive activation of STAT5 and STAT3 Other mutations in JAKs, especially V617F homologous JAK1 mutations, are associated with T-adult acute lymphoblastoid leukaemia (T-ALL) [179, 180]. No JAK or STAT mutation was reported in GBMs.

## Epigenetic alterations in gliomas

### Bmi-1

The polycomb repressor complex 1 component Bmi-1 plays a key role in the maintenance of stemness and self-renewal of cancer stem cells. According to Hayry *et al.,* Bmi-1 is frequently expressed in gliomas, with a significant correlation between the frequency of Bmi-1 immunoreactive tumour cells and poor survival in grades II–III oligoastrocytomas [181].

Also, its presence is required for malignant transformation of NSCs and astrocytes; high levels of Bmi-1 expression facilitate high-grade gliomas *in vivo*, while low expressing Bmi-1 cells initiate or are associated with less malignant glioma [182]. Bmi-1 is considered a promising therapeutic target for stem cell-targeted treatment.

### IDH1, IDH2 and TET2 mutations

Mutations that affect amino acid residue 132 of IDH1 were found in ∼70% of WHO grade II and grade III astrocytomas and oligodendrogliomas and in the secondary glioblastomas developed from these tumours [183]. Isocitrate dehydrogenase 1 mutations might be an early event with more frequent detections in low-grade gliomas. Tumours negative for IDH1 mutations frequently exhibited mutations at the analogous amino acid (R172) of the IDH2 [183]. Isocitrate dehydrogenase mutated tumours had a better outcome than those with wild-type IDH genes. However, this is not the case when, for example, IDH1 mutation is associated with expression of PDGFR alpha, which predicts shorter survival [136].

The mutations are changing the function of IDH enzymes, from rendering isocitrate to α-ketoglutarate; the mutant IDH proteins catalyse the production of D-2-hydroxyglutarate from α-ketoglutarate (α-KG) [173, 184]. The product D-2-hydroxyglutarate inhibits competitively α-KG-dependent enzymes such as TET (Ten Eleven Translocation) hydroxylases and demethylases. In AML, IDH1/2 mutations are also detected and they do correlate with better outcome [185]. Although most low-grade gliomas carry IDH1/2 mutations, some do not, and for those, it was recently found that TET2 miscoding mutations were not detected, but 14% of the cases had methylation of the TET2 promoter [186]. None of the IDH1/2 mutated gliomas had methylation of the TET2 promoter. Thus, like in haematological malignancies, absence of TET2 expression appears to be mutually exclusive with IDH1/2 mutations, and this makes sense as the product of mutated IDH1/2 is an inhibitor of TET2.

Interestingly, bi-allelic inactivation of TET2 leads in the haematopoietic system to enhanced self-renewal of HSCs [187]. Ten-Eleven-Translocation 2 mutations are an independent positive prognostic factor in myelodysplastic syndromes [187].

Ten-Eleven-Translocation enzymes catalyse the conversion of 5-methylcytosine into 5-hydroxymethylcytosine, which might be very important for DNA demethylation and epigenetic regulation. Overall, it is tempting to speculate that, like in the haematopoietic system, absence of TET2 activity, either by IDH1/2 mutations or by promoter methylation, promotes self-renewal of tumour-initiating cells for low-grade diffuse gliomas.

Recurrent mutations affecting two critical amino acids (K27 and G34) of histone H3.3 are found in approximately one-third of paediatric GBM [188], thus emphasizing the connection between chromatin remodelling, structure and glioblastoma. The histone H3.3 variant is normally enriched in chromatin regions of high turnover such as telomeres and is associated with chaperone proteins such as DAXX (death-domain associated protein) and ATRX (alpha-thalassaemia/mental retardation syndrome X-linked), which themselves are mutated in 31% of paediatric GBM and in several cancers including pancreatic neuroendocrine tumours and AML [188, 189]. Such mutations define epigenetic subgroups of GBM with distinct global methylation patterns and are mutually exclusive with IDH1 mutations [190]. Of great interest, the different H3.3 mutations give rise to GBM in separate anatomic compartments, with differential regulation of transcription factors [190].

Overall TET2 and IDH1/2 mutations are being mainly described in haematopoietic and brain cancers, but recently, it was shown that 5-hydroxymethylcytosine depletion was detected in those and other cancers such as squamous cell lung cancer, in the absence of TET2 and IDH mutations [149]. Thus, it is possible that depletion of 5-hydroxymethylcytosine and possibly hypermethylation of certain DNA regions might be a more common cancer feature, and other mutations need to be discovered to explain these observations in other cancers.

## Concluding remarks

We may be entering a new phase in cancer research based on the cancer stem-cell paradigm and we expect that in the near future the actual role of cancer stem cells in malignancy to be unravelled. The proposed difference between cancer-initiating cells and tumour propagation cells should be further explored through a genomic/proteomic panel of biomarkers. Fundamental research would probably unravel in the near future more common characteristics between brain tumours and haematopoietic cancers, especially parallels between low-grade gliomas and chronic cancers or between high-grade gliomas and secondary acute leukaemias. Treatment of haematopoietic cancers extensively benefited from the understanding of haematopoiesis *via* retrospective assays and detailed sorting procedures, identification of initiating events and targeting them with specific inhibitors. Moreover, bone marrow transplant approaches evolved along with detailed cellular and molecular monitoring, taking advantage of the possibilities to suppress host haematopoiesis and reconstitute it in a long-term fashion by transplant. However, certain secondary as well as *de novo* leukaemias remain highly resistant to treatment and resemble high-grade gliomas, without a clear molecular understanding of the mechanisms behind blast expansion or effective approaches to arrest proliferation of such blasts. Several players such as TET2 or IDH mutations appear to be common. Basic research will be essential to delineate the links between signalling and epigenetics changes that drive malignant proliferation. Genomic studies of individual clones will chart the clonal architecture and heterogeneity of these tumours. Therapy will directly depend on discovering the initiating events, which are still to be determined for gliomas, being able to define events acquired during progression, and the pressures driving the entire process. Given their rampant proliferation, as well as their resistance to current treatments and single agents, new combination therapies would eventually be required for GBMs that would be tailored for those active pathways driving proliferation and taking into account the clonal architecture and actual mutations for each patient.

## Note added in proof

While this review was submitted, a new study reported that glioma cancer stem cells exhibit high levels of nuclear activated STAT3 and NFkB, that interact and are bound to the promoter of the Notch gene, leading to induction of Notch and subsequently Notch-dependent gene expression in GCSCs [200].
